# Correction: Computational gene expression analysis reveals distinct molecular subgroups of T-cell prolymphocytic leukemia

**DOI:** 10.1371/journal.pone.0315260

**Published:** 2024-12-05

**Authors:** The PLOS One Staff

There are errors in the author affiliations. The correct affiliations are as follows:

Nathan Mikhaylenko^1^, Linus Wahnschaffe^2,3,4^, Marco Herling^2,3,4,5^, Ingo Roeder^1,6^, Michael Seifert^1,6^

**1** Institute for Medical Informatics and Biometry (IMB), Carl Gustav Carus Faculty of Medicine, Technische Universität Dresden, Dresden, Germany, **2** Department I of Internal Medicine, Center for Integrated Oncology (CIO), Aachen-Bonn-Cologne-Duesseldorf, University of Cologne, Cologne, Germany, **3** Excellence Cluster for Cellular Stress Response and Aging-Associated Diseases (CECAD), University of Cologne, Cologne, Germany, **4** Center for Molecular Medicine Cologne (CMMC), University of Cologne, Cologne, Germany, **5** Department of Hematology and Cellular Therapy, University of Leipzig, Leipzig, Germany, **6** National Center for Tumor Diseases (NCT), Dresden, Germany, German Cancer Research Center (DKFZ), Heidelberg, Germany, Faculty of Medicine and University Hospital Carl Gustav Carus, Technische Universität Dresden, Dresden, Germany, Helmholtz-Zentrum Dresden—Rossendorf (HZDR), Dresden, Germany

In the subsection of Introduction, a Figure citation is omitted from the third sentence of the first paragraph. The correct sentence is: Each of these 13 patient-specific RNA-seq samples always had the strongest correlation with the gene expression profile of its corresponding patient-specific microarray sample (S10 Fig).

The publisher apologizes for the errors.
