# Supplementary material for: Computational gene expression analysis reveals distinct molecular subgroups of T-cell prolymphocytic leukemia
Source: PLoS One. 2022 Sep 21;17(9):e0274463. doi: 10.1371/journal.pone.0274463 (PMC9491575; doi:10.1371/journal.pone.0274463)
Supplement: S1 Fig — (PDF) [file pone.0274463.s001.pdf]

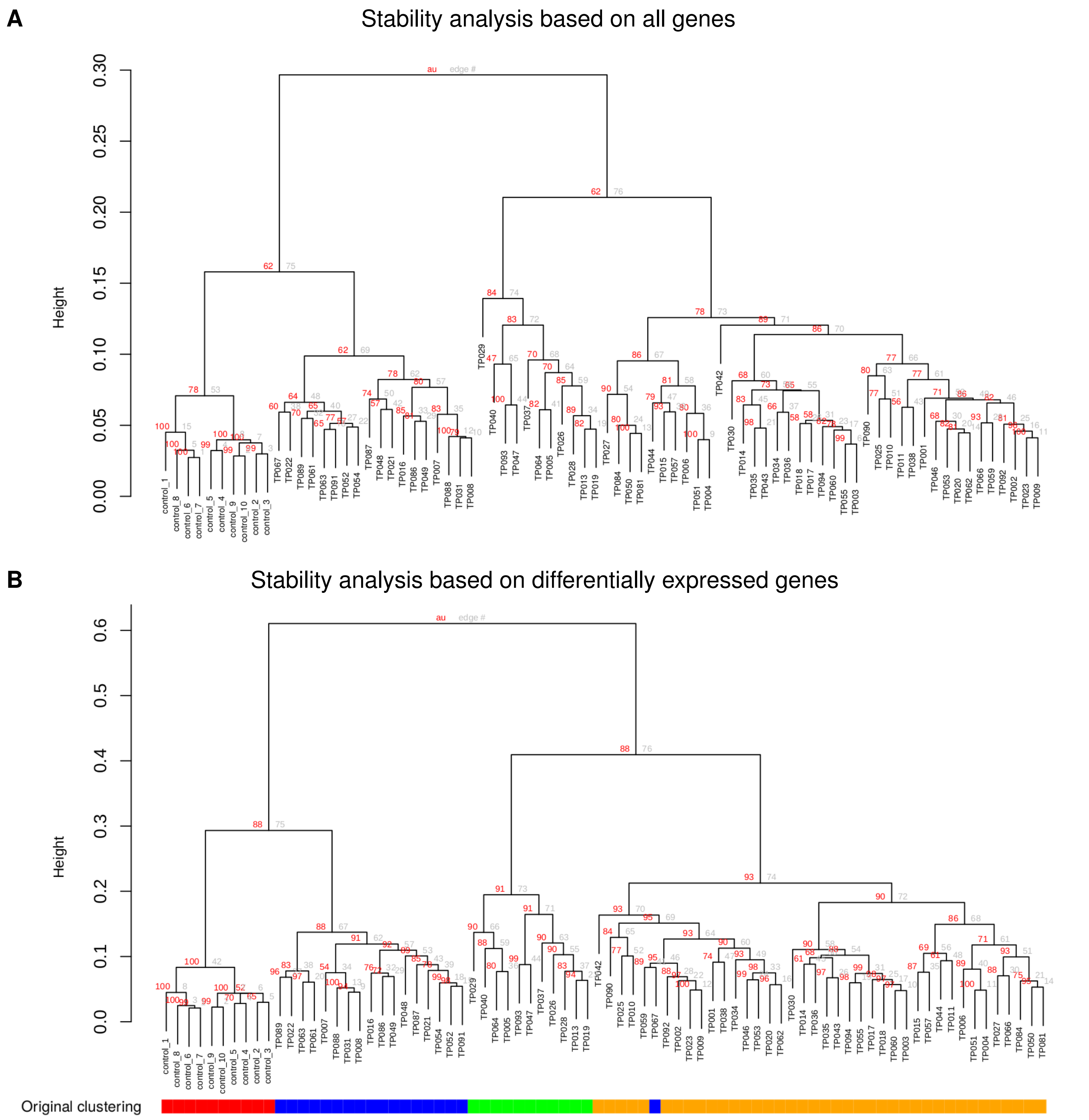

S1 Figure: Stability analysis of the hierarchical clustering of the 68 T-PLL patients and the 10 normal reference controls. The R package pvclust was used to compute approximately unbiased bootstrap values (AU values) to analyze the stability of the clusters by averaging the results of 10,000 bootstrap runs as described in the methods section of the main manuscript. AU values are shown in red above each subtree. The grey number above each subtree specifies its corresponding edge number. The AU value reflects how often the same subtree occurred during the bootstrap analysis. The greater the AU value (max. value 100), the more stable a subtree. **A**, Stability analysis of the column-dendrogram in Figure 1 of the main text based on all 17,970 measured genes. The control samples and the three revealed T-PLL subgroups are represented by the following subtrees: control (edge 53, AU = 78), SG1 (edge 69, AU = 62), SG2 (edge 74, AU = 84), and SG3 (edge 73, AU = 78). **B**, Stability analysis of the column-dendrogram in S6 Figure based on all 5,858 differentially expressed genes. The original subgroup assignment of each sample is shown by a colored bar below the dendrogram. All samples, except TP067, were again assigned to the same subgroup for the reduced gene set. Subgroup stabilities are clearly increased compared to the stability analysis based on all 17,970 genes in subpanel A. The subgroups reach the following stability values: control (edge 42, AU = 100), SG1 minus TP067 sample (edge 67, AU = 88), SG2 (edge 73, AU = 91), and SG3 plus TP067 sample (edge 74, AU = 93).
