# Supplementary material for: Computational gene expression analysis reveals distinct molecular subgroups of T-cell prolymphocytic leukemia
Source: PLoS One. 2022 Sep 21;17(9):e0274463. doi: 10.1371/journal.pone.0274463 (PMC9491575; doi:10.1371/journal.pone.0274463)
Supplement: S2 Fig — (PDF) [file pone.0274463.s002.pdf]

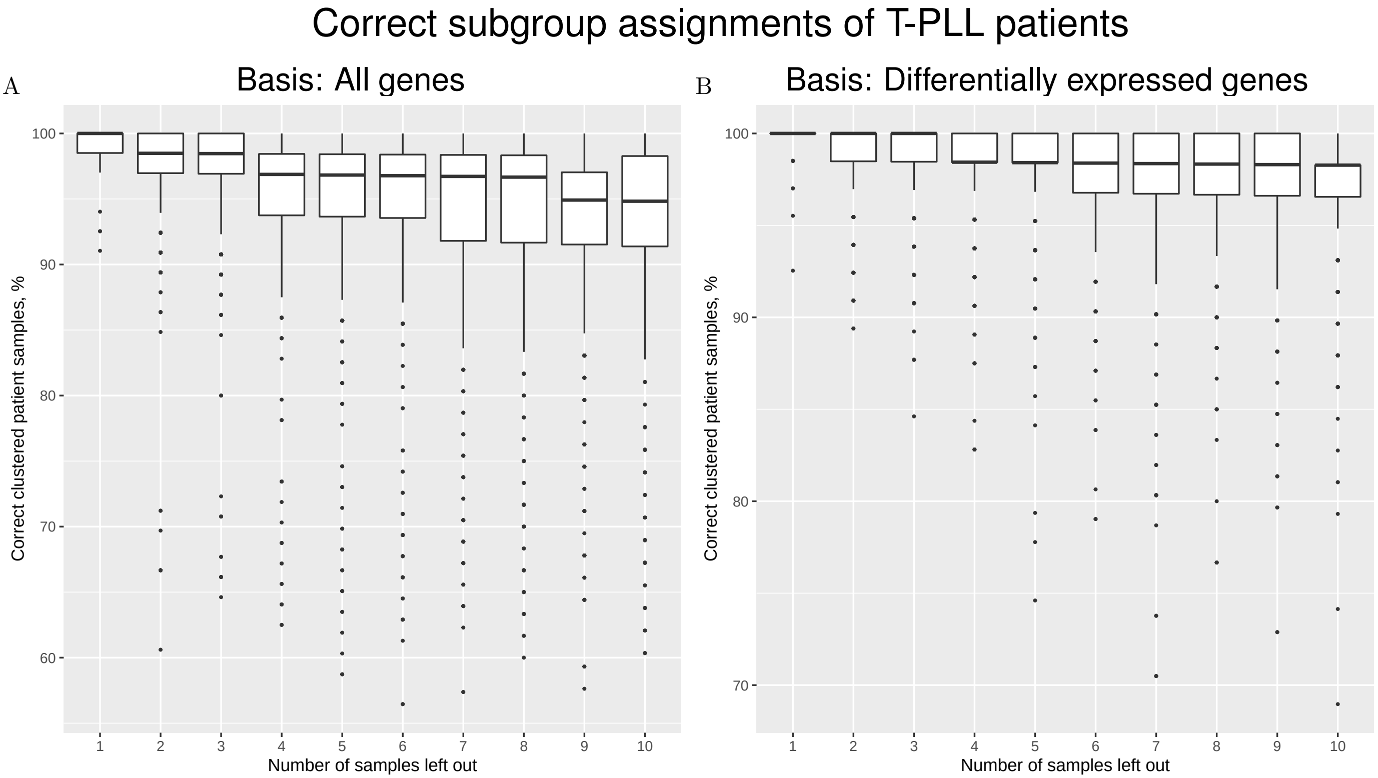

S2 Figure: Stability analysis of T-PLL subgroup assignments. Random subsets of the 68 T-PLL patient samples in combination with the 10 normal control references were considered to repeat the hierarchical clustering of the corresponding gene expression profiles. The four resulting major clusters of each repeated hierarchical clustering were paired to the initially obtained clusters (control, SG1, SG2, SG3) based on the majority of overlapping samples, which enabled to determine the number of correctly clustered samples per run. For the leave-one-out runs, the boxplot is based on the results for removing each of the 68 T-PLL samples individually. For random removals of two up to ten patients, the boxplots represent the results of 1000 random repetitions. **A**, Boxplots of correctly classified T-PLL patients for repeated hierarchical clusterings based on all 17,970 measured genes. **B**, Boxplots of correctly classified T-PLL patients for repeated hierarchical clusterings based on all 5,858 differentially expressed genes.
