# Supplementary material for: Computational gene expression analysis reveals distinct molecular subgroups of T-cell prolymphocytic leukemia
Source: PLoS One. 2022 Sep 21;17(9):e0274463. doi: 10.1371/journal.pone.0274463 (PMC9491575; doi:10.1371/journal.pone.0274463)
Supplement: S3 Fig — (PDF) [file pone.0274463.s003.pdf]

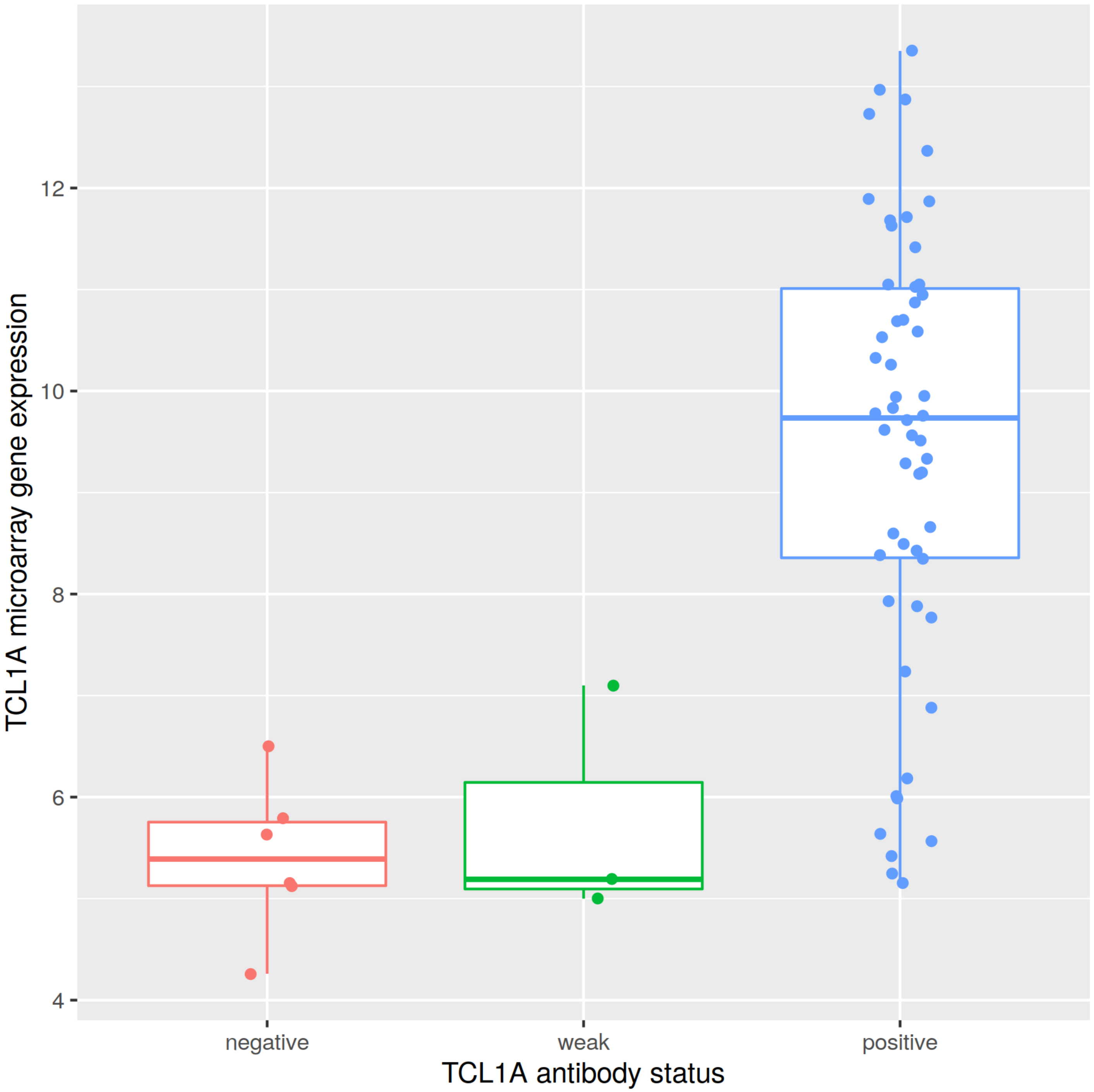


**S3 Figure:** Associations between TCL1A protein expression and *TCL1A* gene expression of T-PLL patients. TCL1A protein expression of each individual T-PLL patient was measured in Schrader et al. (2018) by flow cytometry in combination with antibodies against human TCL1A (clone 1-21) from BioLegend. The measured TCL1A protein expression was further assigned to one of three TCL1A protein expression states by Schrader et al. (2018) (negative: no TCL1A protein expression detected, weak: weak TCL1A protein expression detected, positive: TCL1A expression detected). Boxplots represent the corresponding mRNA expression levels of the *TCL1A* gene of individual T-PLL patients for the three TCL1A protein expression status groups. A significant association between the protein expression status and the corresponding *TCL1A* gene expression levels exist (Kruskal-Wallis test: $p = 0.0001238$). The median *TCL1A* expression levels of the T-PLL patients with positive TCL1A protein expression status are significantly greater than those of patients in the TCL1A negative status group (Wilcoxon rank sum test: $p = 0.0004$) or than those of patients in the TCL1A weak status group (Wilcoxon rank sum test: $p = 0.01$).
