# Supplementary material for: Computational gene expression analysis reveals distinct molecular subgroups of T-cell prolymphocytic leukemia
Source: PLoS One. 2022 Sep 21;17(9):e0274463. doi: 10.1371/journal.pone.0274463 (PMC9491575; doi:10.1371/journal.pone.0274463)
Supplement: S4 Fig — (PDF) [file pone.0274463.s004.pdf]

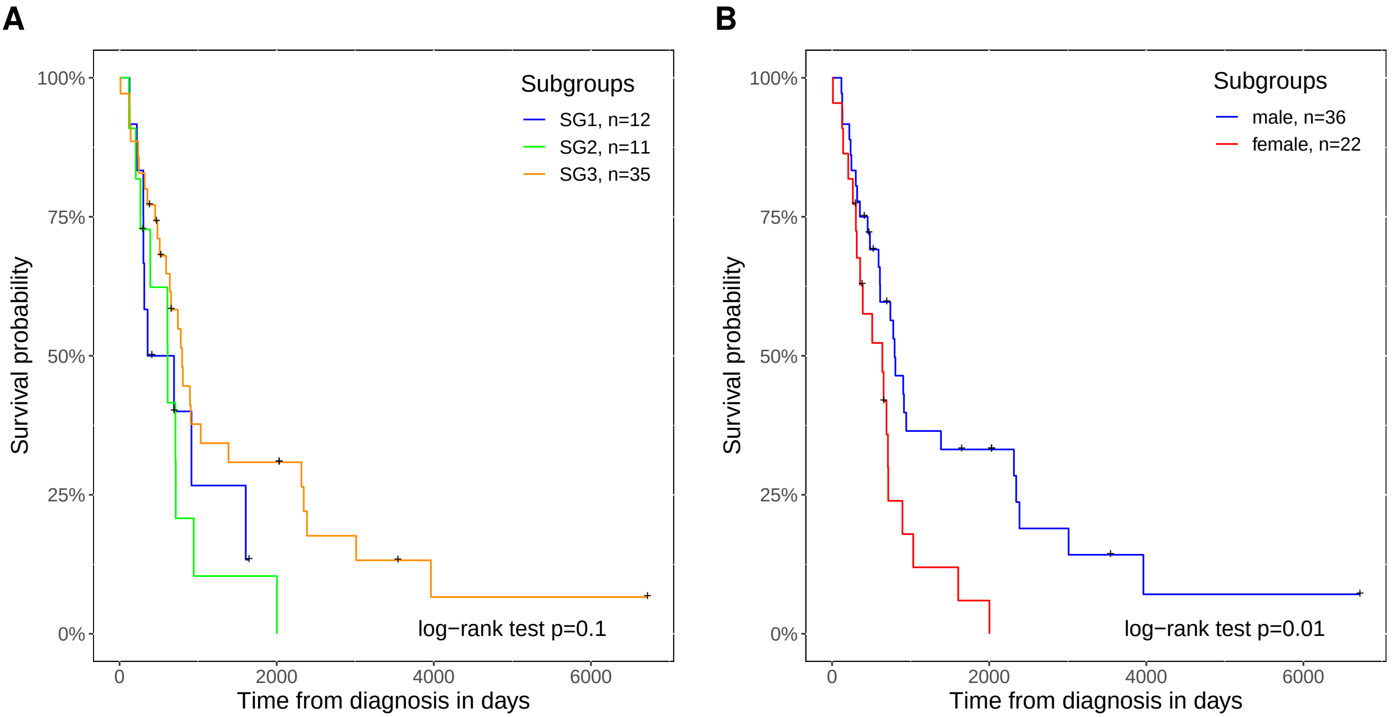

S3 Figure: Kaplan-Meier analysis of T-PLL patients. **A,** Overall survival in days from initial diagnosis for T-PLL patients of the three revealed T-PLL gene expression subgroups SG1, SG2, and SG3. **B,** Overall survival in days from initial diagnosis distinguishing male and female patients.
