# Supplementary material for: Computational gene expression analysis reveals distinct molecular subgroups of T-cell prolymphocytic leukemia
Source: PLoS One. 2022 Sep 21;17(9):e0274463. doi: 10.1371/journal.pone.0274463 (PMC9491575; doi:10.1371/journal.pone.0274463)
Supplement: S5 Fig — (PDF) [file pone.0274463.s005.pdf]

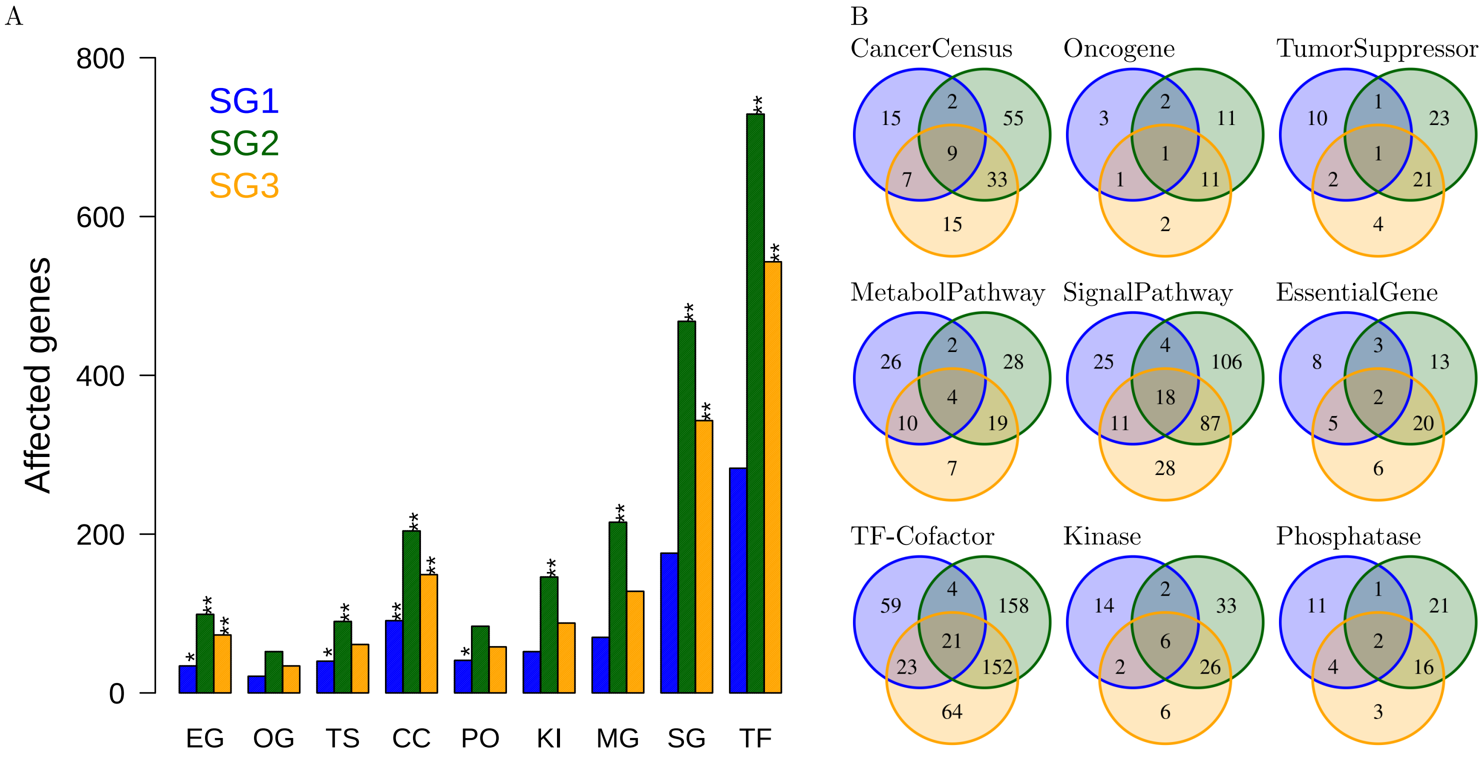

S4 Figure: Gene annotation analysis of differentially expressed genes of T-PLL subgroups. **A,** Enrichment analysis of annotation groups for differentially expressed genes predicted in each of the three T-PLL subgroups SG1 (blue), SG2 (green), and SG3 (orange) (diff. genes: S4 Table $q \leq 0.05$, annotations: S5 Table). Up- and down-regulated genes of each subgroup were jointly analyzed. Enrichment was tested by Fisher's exact test and adjusted for multiple testing: essential genes (EG), oncogenes (OG), tumor suppressor genes (TS), cancer census genes (CC), phosphatases (PO), kinases (KI), metabolic pathway genes (MG), cancer-related signaling pathway genes (SG), and transcription factors/cofactors (TF). Significant enrichment of an individual category is highlighted by '*' for a q-value $\leq$ 0.05 and by '**' for a q-value $\leq$ 0.01. **B,** Venn diagrams visualizing the overlap of up-regulated genes for the specific annotation groups across the three T-PLL subgroups: SG1 (blue), SG2 (green), and SG3 (orange) (diff. genes: S4 Table $q \leq 0.05$, annotations: S5 Table). Oncogenes and tumor suppressor genes have the smallest overlap between SG1 and the two other subgroups SG2 and SG3. Transcription factors/cofactors have the greatest overlap between all subgroups. Proportions of common genes shared between all T-PLL subgroups for all annotation groups: cancer census genes - 6.6%, oncogenes - 3%, tumor suppressor genes - 1.6%, metabolic pathway genes - 4%, signaling pathway genes - 6.4%, essential genes - 3.5%, transcription factors/cofactors - 4%, kinases - 6.7%, phosphatases - 3.4%.
