# Supplementary material for: Computational gene expression analysis reveals distinct molecular subgroups of T-cell prolymphocytic leukemia
Source: PLoS One. 2022 Sep 21;17(9):e0274463. doi: 10.1371/journal.pone.0274463 (PMC9491575; doi:10.1371/journal.pone.0274463)
Supplement: S6 Fig — (PDF) [file pone.0274463.s006.pdf]

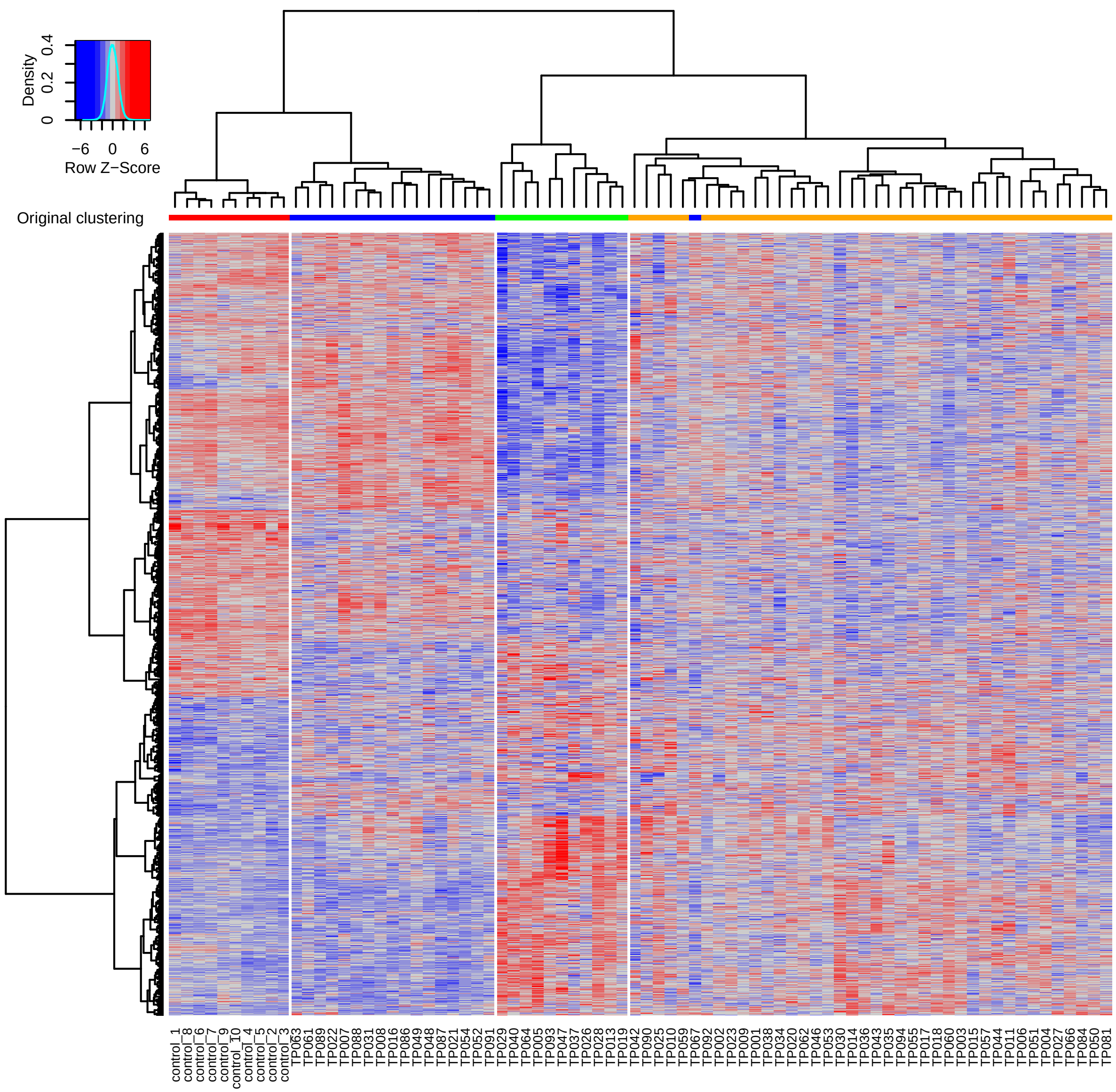

S5 Figure: Heatmap-based clustering of T-PLL and normal control expression profiles based on all differentially expressed genes that were identified in the pairwise comparisons of each of the three T-PLL subgroups to the normal control references (S4 Table, $q \leq 0.05$). Z-score-scaled expression measurements of the corresponding 5,858 genes are shown in the heatmap for each sample highlighting reduced (blue), unchanged (grey), and increased (red) expression of each gene in a specific sample in comparison to the other samples. T-PLL and normal control samples (columns) were hierarchically clustered based on the similarity of their expression profiles and their corresponding gene-specific expression values are visualized (rows). The column dendrogram above the heatmap is very similar to that in Figure 1 of the main manuscript, which considered all 17,970 genes. The original cluster assignments of the samples from the hierarchical clustering in Figure 1 are visualized below the column dendrogram: healthy control (red) and three T-PLL subclusters (blue: SG1, green: SG2, orange: SG3). Only sample TP067 switched from SG1 to SG3. Individual sample names are shown in the corresponding columns below the heatmap. The dendrogram left to the heatmap represents the clustering of the differentially expressed genes.
