# Supplementary material for: Computational gene expression analysis reveals distinct molecular subgroups of T-cell prolymphocytic leukemia
Source: PLoS One. 2022 Sep 21;17(9):e0274463. doi: 10.1371/journal.pone.0274463 (PMC9491575; doi:10.1371/journal.pone.0274463)
Supplement: S7 Fig — (PDF) [file pone.0274463.s007.pdf]

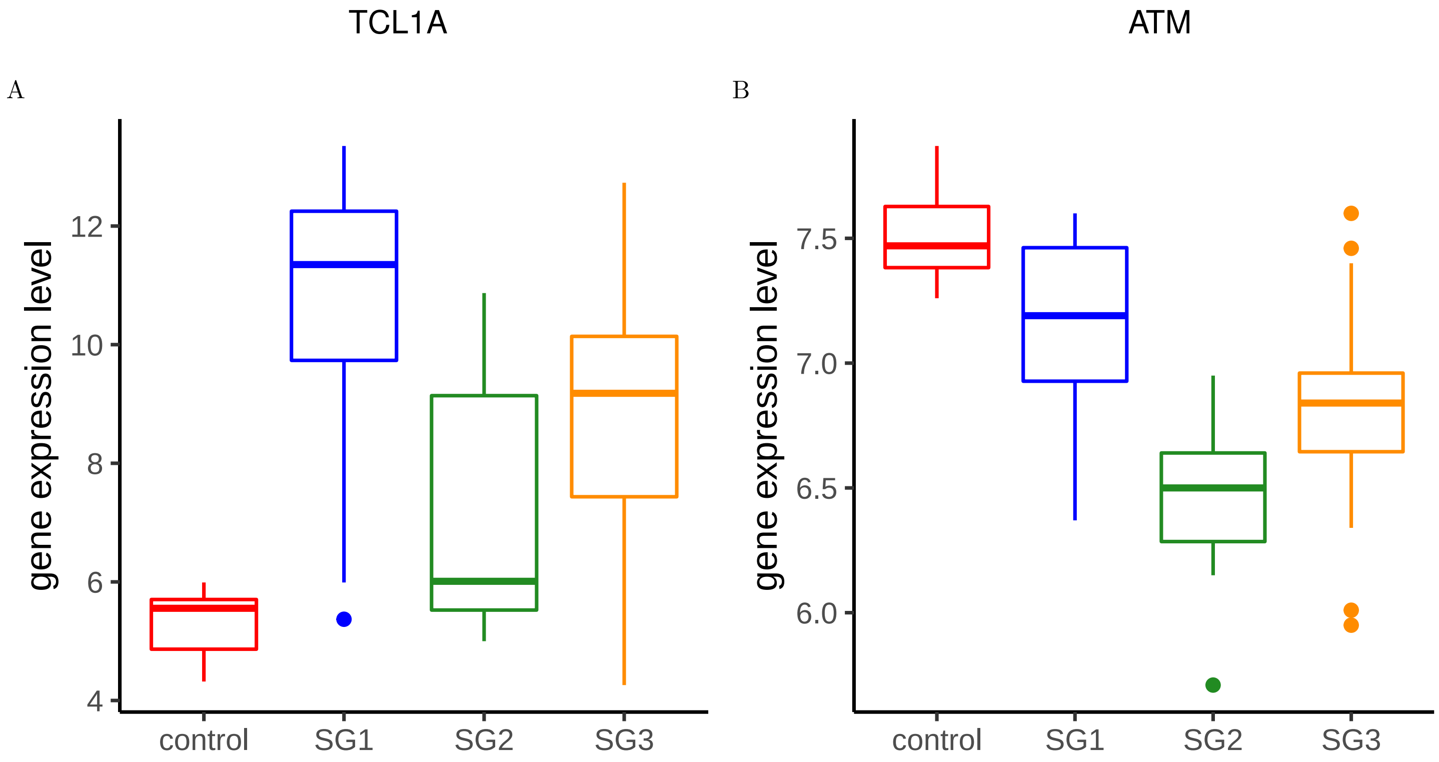

S6 Figure: Boxplots of absolute expression levels of *TCL1A* (subpanel A) and *ATM* (subpanel B) for the normal control references and for each of the three revealed T-PLL subgroups. Corresponding normalized expression values are contained in S2 Table. Results of the pairwise statistical gene expression analysis comparing the gene-specific expression levels of each T-PLL subgroup to normal controls are provided in S4 Table.
