# Supplementary material for: Computational gene expression analysis reveals distinct molecular subgroups of T-cell prolymphocytic leukemia
Source: PLoS One. 2022 Sep 21;17(9):e0274463. doi: 10.1371/journal.pone.0274463 (PMC9491575; doi:10.1371/journal.pone.0274463)
Supplement: S8 Fig — (PDF) [file pone.0274463.s008.pdf]

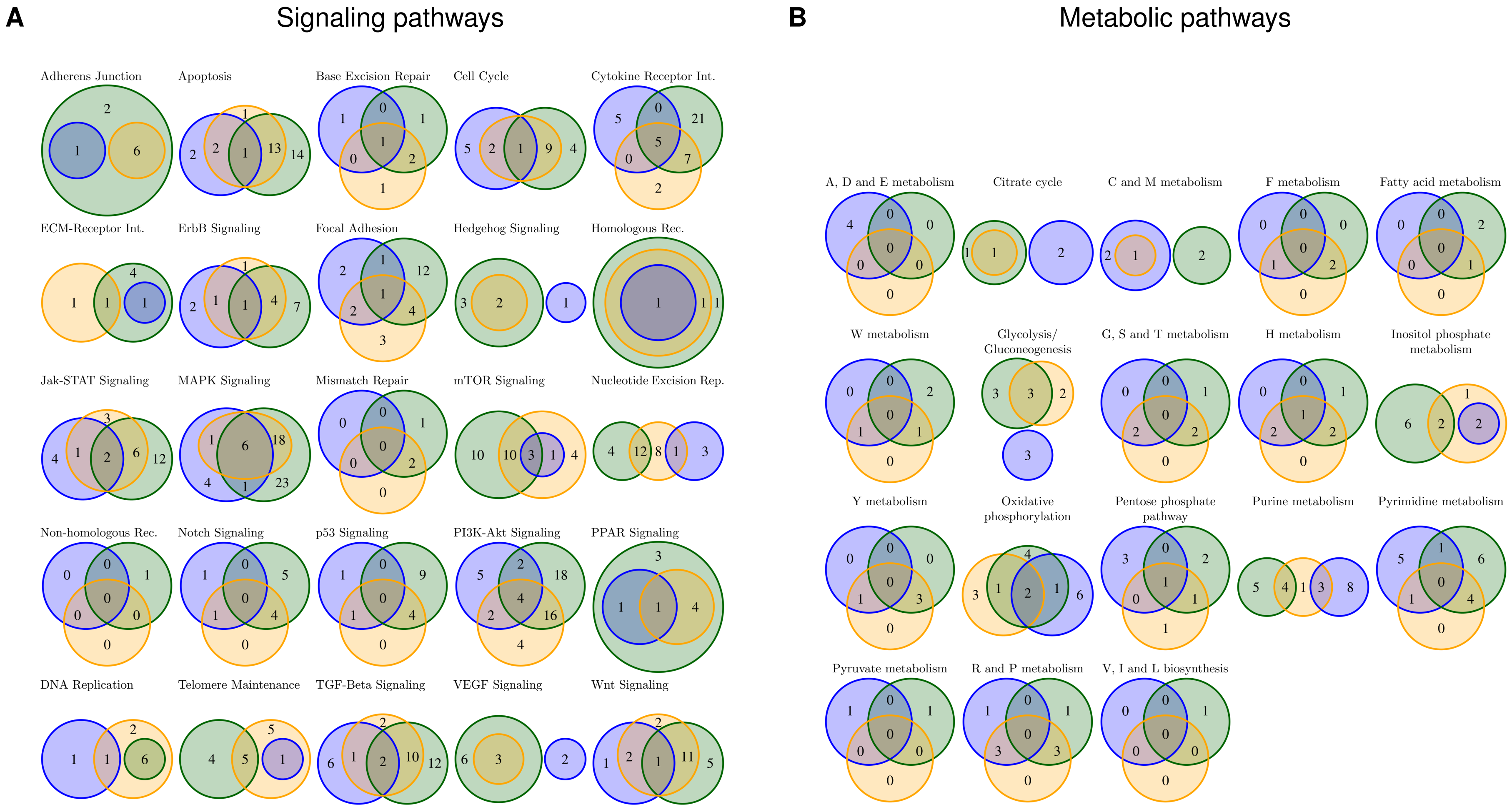

S7 Figure: Venn diagrams analyzing the overlap of up-regulated signaling pathway (subpanel A) or metabolic pathway (subpanel B) genes. All significantly up-regulated pathway genes with a q-value ≤ 0.05 were considered (genes: S4 Table, pathway annotations: S5 Table). Colored circles represent the T-PLL subgroups: SG1 (blue), SG2 (green), and SG3 (orange).
