# Supplementary material for: Computational gene expression analysis reveals distinct molecular subgroups of T-cell prolymphocytic leukemia
Source: PLoS One. 2022 Sep 21;17(9):e0274463. doi: 10.1371/journal.pone.0274463 (PMC9491575; doi:10.1371/journal.pone.0274463)
Supplement: S9 Fig — (PDF) [file pone.0274463.s009.pdf]

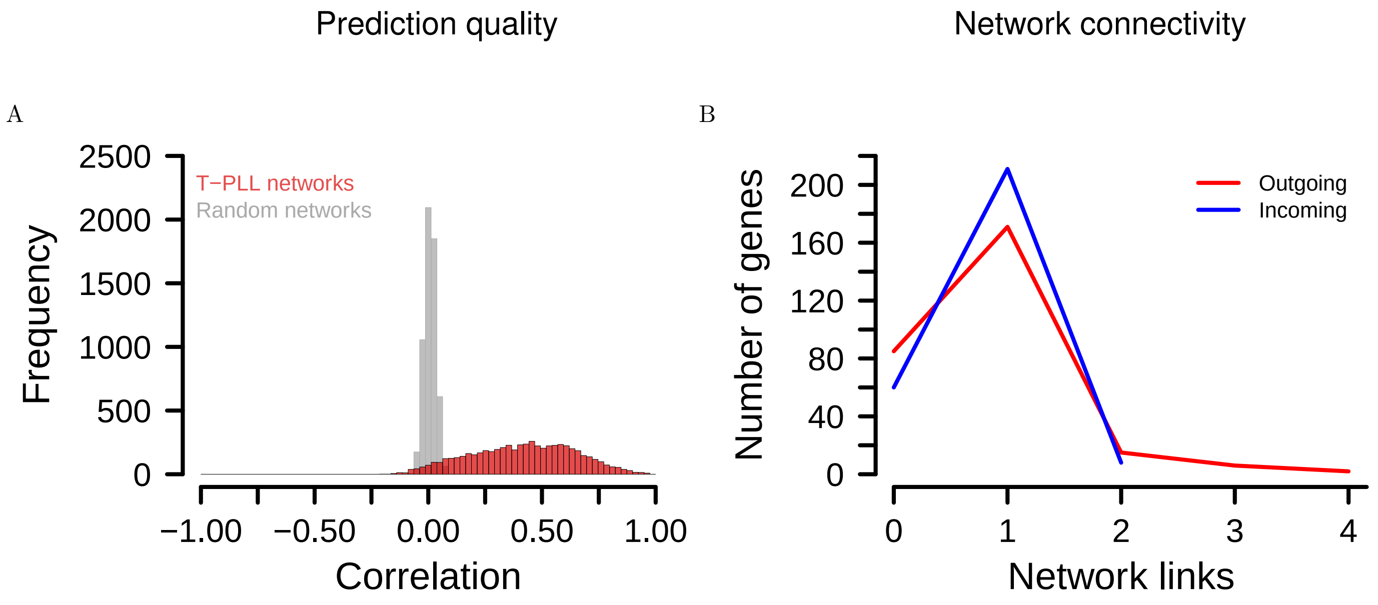

S8 Figure: Prediction quality and connectivity of the T-PLL gene expression signature-specific gene regulatory networks derived for the 5,858 differentially expressed genes. **A**, Distribution of average correlations representing the prediction quality of individual gene expression levels for the 100 learned networks (red) and their corresponding random networks of same complexity (grey). Pearson correlations between network-based predicted and originally measured expression levels of individual genes were determined for each independent network-specific test set. Only network links between genes with a network link q-value $\leq$ 0.01 were considered for the prediction of gene expression levels. T-PLL-specific networks reached significantly better predictions for individual genes (red) than random networks of same complexity (grey) (paired Wilcoxon signed rank test: $p < 2.2 \cdot 10^{-16}$). **B**, Number of genes with a specific number of outgoing and incoming network links that were present in at least 75 of the 100 learned networks at a network link q-value cutoff of 0.01.
