# Supplementary material for: Computational gene expression analysis reveals distinct molecular subgroups of T-cell prolymphocytic leukemia
Source: PLoS One. 2022 Sep 21;17(9):e0274463. doi: 10.1371/journal.pone.0274463 (PMC9491575; doi:10.1371/journal.pone.0274463)
Supplement: S10 Fig — (PDF) [file pone.0274463.s010.pdf]

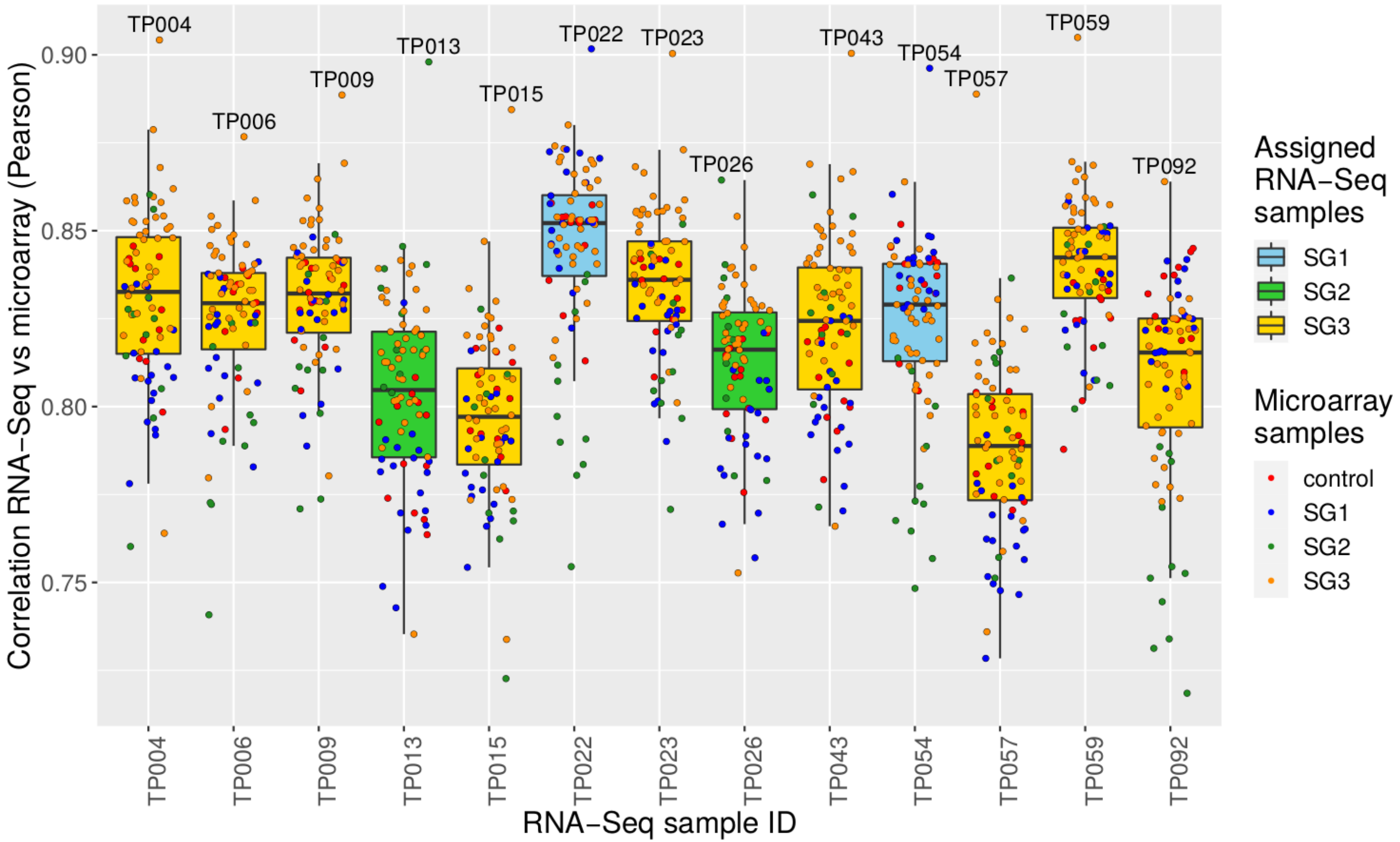

S9 Figure: Boxplots of pairwise correlations between gene expression profiles of T-PLL samples measured by microarray and RNA-seq. Pearson correlations of gene expression profiles were computed for each of the 13 RNA-seq T-PLL patient samples in relation to each microarray T-PLL and control sample of our main study. The color of the box represents the subgroup that was assigned to the RNA-seq sample based on the label of the microarray sample that had the strongest positive correlation to this RNA-seq sample. The obtained pairwise correlations are additionally visualized by the corresponding dots in the area of each boxplot. The dot with the highest correlation is labeled by the corresponding sample name. Each RNA-seq sample of the 13 patients was assigned to its corresponding microarray sample of the underlying patient. The initially assigned subgroups of these patients did not change.
