# Supplementary material for: Computational gene expression analysis reveals distinct molecular subgroups of T-cell prolymphocytic leukemia
Source: PLoS One. 2022 Sep 21;17(9):e0274463. doi: 10.1371/journal.pone.0274463 (PMC9491575; doi:10.1371/journal.pone.0274463)
Supplement: S11 Fig — (PDF) [file pone.0274463.s011.pdf]

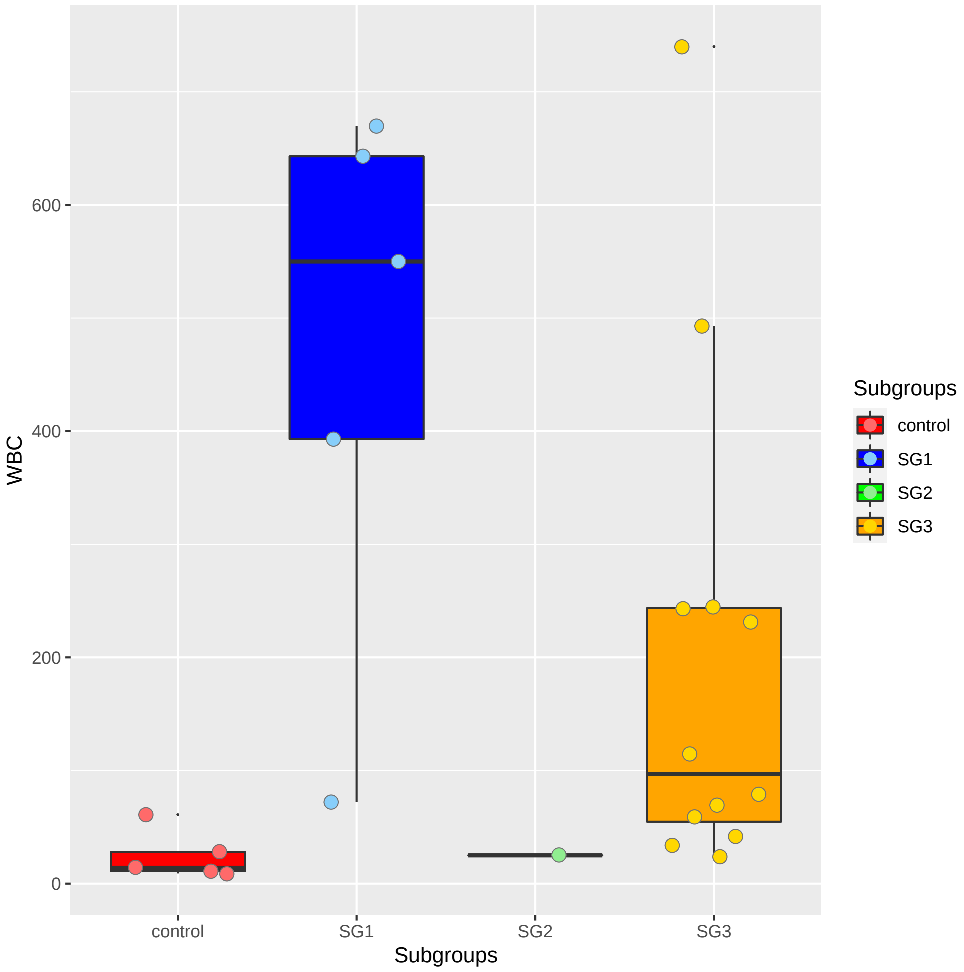

S10 Figure: Boxplots of white blood cell counts (WBC) for T-PLL patients from Erkeland *et al.* (2022) classified by their similarity to our three revealed T-PLL subgroups and our normal controls. The five T-PLL patients that were assigned to the normal control group had clearly less white blood cell counts than patients that were assigned to SG1 or SG3 (ANOVA without SG2: $p = 0.0086$).
