# Supplementary material for: Computational gene expression analysis reveals distinct molecular subgroups of T-cell prolymphocytic leukemia
Source: PLoS One. 2022 Sep 21;17(9):e0274463. doi: 10.1371/journal.pone.0274463 (PMC9491575; doi:10.1371/journal.pone.0274463)
Supplement: S12 Fig — (PDF) [file pone.0274463.s012.pdf]

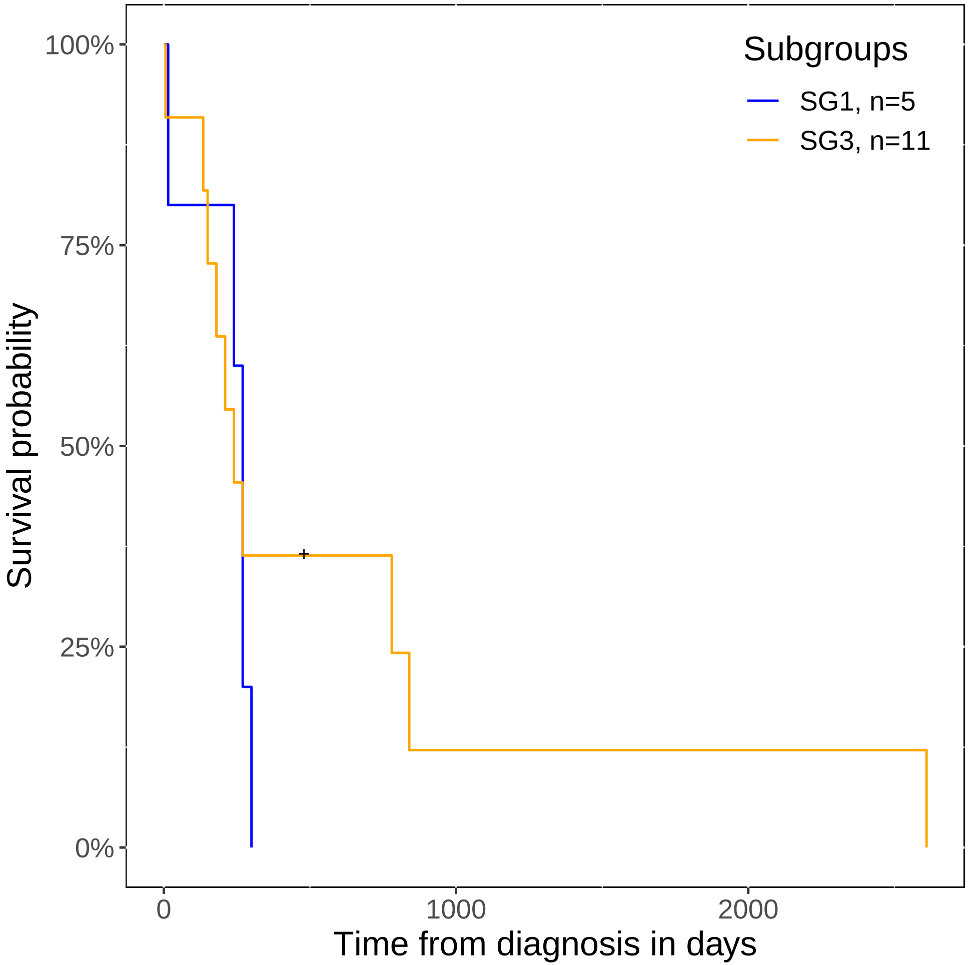

S11 Figure: Kaplan-Meier curves of T-PLL patients from Erkeland *et al.* (2022) that were classified to belong to our revealed SG1 or SG3 subgroup.
