# Supplementary material for: Computational gene expression analysis reveals distinct molecular subgroups of T-cell prolymphocytic leukemia
Source: PLoS One. 2022 Sep 21;17(9):e0274463. doi: 10.1371/journal.pone.0274463 (PMC9491575; doi:10.1371/journal.pone.0274463)
Supplement: S1 Appendix — (PDF) [file pone.0274463.s022.pdf]

Computational gene expression analysis reveals distinct molecular subgroups of T-cell prolymphocytic leukemia

S1 Appendix: Literature analysis of potential major regulators

We learned T-PLL-specific gene regulatory networks that were associated with the observed expression differences between the three T-PLL subgroups. These networks also included 16 gene modules that represented 41 genes with an increased connectivity to other differentially expressed genes (see Table 1 in main manuscript). We performed an in-depth literature analysis to determine known cellular functions of these genes in relation to cancer. These functions in combination with the characteristic subgroup-specific gene expression patterns contribute to a better characterization of similarities and differences between the three T-PLL subgroups.

- *GP9* and *TUBB1* were down-regulated in all three T-PLL subgroups in comparison to normal controls. *GP9* encodes for a N-glycan, which is involved in the regulation of interactions between cancer cells and microenvironment, and has been reported as a biomarker candidate for breast cancer [Terkelsen et al., 2018]. *TUBB1* encodes a beta tubulin protein that is part of microtubules [Safran et al., 2010]. A knockdown of *TUBB1* has led to a reduction of apoptosis after DNA damage [Matsumura et al., 2019].

- *FFAR2* and *CMTM2* were up-regulated in all three T-PLL subgroups in comparison to normal controls. *FFAR2* encodes a G-protein coupled fatty acid receptor [Safran et al., 2010] and increased *FFAR2* expression has been associated with reduced leukemic cell proliferation [Bindels et al., 2017]. *CMTM2* encodes a chemokine-like factor [Safran et al., 2010] and a downregulation of *CMTM2* has been associated with invasion and migration of hepatocellular carcinoma cells [Zhang et al., 2020].

- Considering genes that were unchanged in SG1 but up-regulated in SG2 and SG3 in comparison to normal controls, the chemokine *CXCL8* has been found to promote survival and proliferation of acute myeloid leukemia cells via PI3K/Akt signaling [Cheng et al., 2019]. *G0S2* has been reported to suppress oncogenic transformation by repressing a *MYC*-regulated transcriptional program [Kitareewan et al., 2008, Yim et al., 2016]. A knockdown of *CA1* has been reported to inhibit growth of diffuse large B cell lymphoma and *CA1* can promote chemotherapy resistance via NF-κB and STAT3 signaling [Feng et al., 2020]. *PPP1R15A* has been reported to be involved in the regulation of DNA damage-induced apoptosis [Adler et al., 1999]. Loss of *IGFBPL1* expression has been reported to promote esophageal cancer growth, whereas expression of *IGFBPL1* has been found to inhibit proliferation and to induce apoptosis and G1/S phase arrest [Liu et al., 2020].

- Considering genes that were unchanged in SG1 but down-regulated in SG2 and SG3, *GIMAP1* has been shown to be critical to maintain the peripheral T-cell pool in mice [Datta et al., 2017]. Generally, *GIMAP* genes are known to be expressed in hematopoietic stem cells and mature T cells and downregulation of *GIMAP* genes is known during immature stages of thymocyte differentiation [Liau et al., 2017].

- Considering genes that were exclusively down-regulated in SG1 but unchanged in SG2 and SG3 in comparison to normal controls, *LCN2* has been reported to be involved in epithelial to mesenchymal transition and invasion of breast cancer [Yang and Moses, 2009]. *LCN2* has also been suggested to be involved in *BCR-ABL*-induced tumorigenesis of chronic myeloid leukemia by inducing apoptosis of normal hematopoietic cells [Lin et al., 2005, Devireddy et al., 2005, Leng et al., 2008, Yang and Moses, 2009]. Expression of amino-terminally deleted *ELANE* isoforms has been reported to reduce myeloid cell clonogenic capacity [Tidwell et al., 2014]. *ELANE* has been found to be highly expressed in leukemia patients and was associated with poor survival [Zhao et al., 2019].

- Considering genes that were exclusively up-regulated in SG2 but unchanged in SG1 and SG3 in comparison to normal controls, *ALPP* has been suggested as a potential biomarker for diagnosis and treatment monitoring of leukemia patients [Patel et al., 1993]. *BLZF1* expression has been associate with survival of hepatocellular carcinoma patients [Huang et al., 2015]. *SLC5A8* has been reported to represent a tumor suppressor that is linked to the Warburg effect [Bhutia et al., 2016]. *USP49* has been found to negatively regulate tumorigenesis and chemoresistance in pancreatic cancer via Akt signaling [Luo et al., 2017]. *IFIT3* has been reported as a potential driver of oral squamous cell carcinoma [Pidugu et al., 2019b], whereas *IFIT2* is known to induce apoptosis [Safran et al., 2010, Pidugu et al., 2019a]. Increased *CXCR2* expression has been associated with lower relapse free survival of acute myeloid leukemia patients [Tang et al., 2020]. The matrix metalloproteinase *MMP25* has been reported to be involved in remodeling of cellular and tissue microenvironments of different cancers [Sohail et al., 2008]. *S100A9* has been found to be involved in the regulation of the differentiation of acute myeloid leukemia cells [Laouedj et al., 2017]. *S100A12* has been found to be involved in myeloid-dependent T-cell suppression [Azzaoui et al., 2016].

- Expression alterations of the three nuclear pore complex interacting proteins that were up-regulated in SG3 but unchanged in SG1 and SG2 (*NPIPB5*, *NPIPB2*, *NPIPB15*) may contribute to a deregulation of essential transport processes between the nucleus and the cytoplasm, which can contribute to cancer development [Köhler and Hurt, 2010, Borden, 2021].

References

[Adler et al., 1999] Adler, H. T., Chinery, R., Wu, D. Y., Kussick, S. J., Payne, J. M., Fornace Jr, A. J., and Tkachuk, D. C. (1999). Leukemic HRX fusion proteins inhibit GADD34-induced apoptosis and associate with the GADD34 and hSNF5/INI1 proteins. *Mol Cell Biol*, 19(10):7050–60.

[Azzaoui et al., 2016] Azzaoui, I., Uhel, F., Rossille, D., Pangault, C., Dulong, J., Le Priol, J., Lamy, T., Houot, R., Le Gouill, S., Cartron, G., Godmer, P., Bouabdallah, K., Milpied, N., Damaj, G., Tarte, K., Fest, T., and Roussel, M. (2016). T-cell defect in diffuse large B-cell lymphomas involves expansion of myeloid-derived suppressor cells. *Blood*, 128(8):1081–1092.

[Bhutia et al., 2016] Bhutia, Y. D., Babu, E., Ramachandran, S., Yang, S., Thangaraju, M., and Ganapathy, V. (2016). SLC transporters as a novel class of tumour suppressors: identity, function and molecular mechanisms. *Biochem J*, 473(9):1113–1124.

[Bindels et al., 2017] Bindels, L. B., Porporato, P. E., Ducastel, S., Sboarina, M., Neyrinck, A. M., Dewulf, E. M., Feron, O., Lestavel, S., Cani, P. D., Staels, B., Sonveaux, P., and Delzenne, N. M. (2017). Ffar2 expression regulates leukaemic cell growth in vivo. *Br J Cancer*, 117(9):1336–1340.

[Borden, 2021] Borden, K. L. B. (2021). The Nuclear Pore Complex and mRNA Export in Cancer. *Cancers*, 13(1):42.

[Cheng et al., 2019] Cheng, J., Li, Y., Liu, S., Jiang, Y., Ma, J., Wan, L., Li, Q., and Pang, T. (2019). CXCL8 derived from mesenchymal stromal cells supports survival and proliferation of acute myeloid leukemia cells through the PI3K/AKT pathway. *FASEB J*, 33(4):4755–4764.

[Datta et al., 2017] Datta, P., Webb, L. M. C., Avdo, I., Pascall, J., and Butcher, G. W. (2017). Survival of mature T cells in the periphery is intrinsically dependent on GIMAP1 in mice. *Eur J Immunol*, 47(1):84–93.

[Devireddy et al., 2005] Devireddy, L. R., Gazin, C., Zhu, X., and Green, M. R. (2005). A cell-surface receptor for lipocalin 24p3 selectively mediates apoptosis and iron uptake. *Cell*, 123(7):1293–305.

[Feng et al., 2020] Feng, Y., Zhong, M., Tang, Y., Liu, X., Liu, Y., Wang, L., and Zhou, H. (2020). The Role and Underlying Mechanism of Exosomal CA1 in Chemotherapy Resistance in Diffuse Large B Cell Lymphoma. *Mol Ther Nucleic Acids*, 21:452–463.

[Huang et al., 2015] Huang, R.-Y., Su, S.-G., Wu, D.-C., Fu, J., and Zeng, X. (2015). BLZF1 expression is of prognostic significance in hepatocellular carcinoma. *Biochem Biophys Res Commun*, 467(3):602–609.

[Kitareewan et al., 2008] Kitareewan, S., Blumen, S., Sekula, D., Bissonnette, R. P., Lamph, W. W., Cui, Q., Gallagher, R., and Dmitrovsky, E. (2008). G0S2 is an all-trans-retinoic acid target gene. *Int J Oncol*, 33(2):397–404.

[Köhler and Hurt, 2010] Köhler, A. and Hurt, E. (2010). Gene regulation by nucleoporins and links to cancer. *Mol Cell*, 38(1):6–15.

[Laouedj et al., 2017] Laouedj, M., Tardif, M. R., Gil, L., Raquil, M.-A., Lachhab, A., Pelletier, M., Tessier, P. A., and Barabé, F. (2017). S100A9 induces differentiation of acute myeloid leukemia cells through TLR4. *Blood*, 129(14):1980–1990.

[Leng et al., 2008] Leng, X., Lin, H., Ding, T., Wang, Y., Wu, Y., Klumpp, S., Sun, T., Zhou, Y., Monaco, P., Belmont, J., Aderem, A., Akira, S., Strong, R., and Arlinghaus, R. (2008). Lipocalin 2 is required for BCR-ABL-induced tumorigenesis. *Oncogene*, 27(47):6110–9.

[Liau et al., 2017] Liau, W. S., Tan, S. H., Ngoc, P. C. T., Wang, C. Q., Tergaonkar, V., Feng, H., Gong, Z., Osato, M., Look, A. T., and Sanda, T. (2017). Aberrant activation of the GIMAP enhancer by oncogenic transcription factors in T-cell acute lymphoblastic leukemia. *Leukemia*, 31(8):1798–1807.

[Lin et al., 2005] Lin, H., Monaco, G., Sun, T., Ling, X., Stephens, C., Xie, S., Belmont, J., and Arlinghaus, R. (2005). Bcr-Abl-mediated suppression of normal hematopoiesis in leukemia. *Oncogene*, 24(20):3246–56.

[Liu et al., 2020] Liu, Y., Zhang, M., He, T., Yang, W., Wang, L., Zhang, L., and Guo, M. (2020). Epigenetic silencing of IGFBPL1 promotes esophageal cancer growth by activating PI3K-AKT signaling. *Clin Epigenetics*, 12:22.

[Luo et al., 2017] Luo, K., Li, Y., Yin, Y., Li, L., Wu, C., Chen, Y., Nowsheen, S., Hu, Q., Zhang, L., Lou, Z., and Yuan, J. (2017). USP49 negatively regulates tumorigenesis and chemoresistance through FKBP51-AKT signaling. *EMBO J*, 36(10):1434–1446.

[Matsumura et al., 2019] Matsumura, T., Nakamura-Ishizu, A., Takaoka, K., Maki, H., Muddineni, S. S. N. A., Wang, C. Q., Suzushima, H., Kawakita, M., Asou, N., Matsuoka, M., Kurokawa, M., Osato, M., and Suda, T. (2019). TUBB1 dysfunction in inherited thrombocytopenia causes genome instability. *Br J Haematol*, 185(5):888–902.

[Patel et al., 1993] Patel, P. S., Adhvaryu, S. G., and Balar, D. B. (1993). Clinical significance of serum total and heat-stable alkaline phosphatase in leukemia patients. *Tumori*, 79(5):352–356.

[Pidugu et al., 2019a] Pidugu, V. K., Pidugu, H. B., Wu, M.-M., Liu, C.-J., and Lee, T.-C. (2019a). Emerging Functions of Human IFIT Proteins in Cancer. *Front Mol Biosci*, 6:148.

[Pidugu et al., 2019b] Pidugu, V. K., Wu, M.-M., Yen, A.-H., Pidugu, H. P., Chang, K.-W., Liu, C.-J., and Lee, T.-C. (2019b). IFIT1 and IFIT3 promote oral squamous cell carcinoma metastasis and contribute to the anti-tumor effect of gefitinib via enhancing p-EGFR recycling. *Oncogene*, 38(17):3232–3247.

[Safran et al., 2010] Safran, M., Dalah, I., Alexander, J., Rosen, N., Stein, T. I., Shmoish, M., Nativ, N., Bahir, I., Doniger, T., Krug, H., et al. (2010). GeneCards Version 3: The human gene integrator. *Database*, 2010:baq020.

[Sohail et al., 2008] Sohail, A., Sun, Q., Zhao, H., Bernardo, M. M., Cho, J.-A., and Fridman, R. (2008). MT4-(MMP17) and MT6-MMP (MMP25), A unique set of membrane-anchored matrix metalloproteinases: properties and expression in cancer. *Cancer Metastasis Rev*, 27(2):289–302.

[Tang et al., 2020] Tang, W., Li, Z., Li, X., and Huo, Z. (2020). High CXCR2 expression predicts poor prognosis in adult patients with acute myeloid leukemia. *Ther Adv Hematol*, 11:2040620720958586.

[Terkelsen et al., 2018] Terkelsen, T., Haakensen, V. D., Saldova, R., Gromov, P., Hansen, M. K., Stöckmann, H., Lingjaerde, O. C., Børresen-Dale, A.-L., Papaleo, E., Helland, A., Rudd, P. M., and Gromova, I. (2018). N-glycan signatures identified in tumor interstitial fluid and serum of breast cancer patients: association with tumor biology and clinical outcome. *Mol Oncol*, 12(6):972–990.

[Tidwell et al., 2014] Tidwell, T., Wechsler, J., Nayak, R. C., Trump, L., Salipante, S. J., Cheng, J. C., Donadieu, J., Glaubach, T., Corey, S. J., Grimes, H. L., Lutzko, C., Cancelas, J. A., and Horwitz, M. S. (2014). Neutropenia-associated ELANE mutations disrupting translation initiation produce novel neutrophil elastase isoforms. *Blood*, 123(4):562–9.

[Yang and Moses, 2009] Yang, J. and Moses, M. A. (2009). Lipocalin 2: A Multifaceted Modulator of Human Cancer. *Cell Cycle*, 8(15):2347–2352.

[Yim et al., 2016] Yim, C. Y., Sekula, D. J., Hever-Jardine, M. P., Liu, X., Warzecha, J. M., Tam, J., Freemantle, S. J., Dmitrovsky, E., and Spinella, M. J. (2016). G0S2 Suppresses Oncogenic Transformation by Repressing a MYC-Regulated Transcriptional Program. *Cancer Res.*, 76(5):1204–1213.

[Zhang et al., 2020] Zhang, S., Tian, R., Bei, C., Zhang, H., Kong, J., Zheng, C., Song, X., Li, D., Tan, H., Zhu, X., and Tan, S. (2020). Down-Regulated CMTM2 Promotes Epithelial-Mesenchymal Transition in Hepatocellular Carcinoma. *Onco Targets Ther*, 13:5731–5741.

[Zhao et al., 2019] Zhao, Y., Si, L., Zhang, W., Huang, W., and Wang, R. (2019). ELANE is highly expressed in leukemia patients and predicts poor survival. *Int J Clin Exp Med*, 12(4):3153–3160.
