# Supplementary material for: Computational gene expression analysis reveals distinct molecular subgroups of T-cell prolymphocytic leukemia
Source: PLoS One. 2022 Sep 21;17(9):e0274463. doi: 10.1371/journal.pone.0274463 (PMC9491575; doi:10.1371/journal.pone.0274463)
Supplement: Attachment — Submitted filename: ResponseToReviewerComments.pdf [file pone.0274463.s023.pdf]

Point-by-Point Response to Journal Requirements and Reviewer Comments

Journal Requirements: When submitting your revision, we need you to address these additional requirements.

1. Please ensure that your manuscript meets PLOS ONE's style requirements, including those for file naming. The PLOS ONE style templates can be found at
https://journals.plos.org/plosone/s/file?id=wjVg/PLOSOne_formatting_sample_main_body.pdf and
https://journals.plos.org/plosone/s/file?id=ba62/PLOSOne_formatting_sample_title_authors_affiliations.pdf

Reply: We checked the provided style templates and made the corresponding changes to the manuscript. We updated the author affiliations. We renamed the supplementary tables.

2. We note that the grant information you provided in the 'Funding Information' and 'Financial Disclosure' sections do not match. When you resubmit, please ensure that you provide the correct grant numbers for the awards you received for your study in the 'Funding Information' section. Please note that funding information should not appear in the Acknowledgments section or other areas of your manuscript. We will only publish funding information present in the Funding Statement section of the online submission form. Please remove any funding-related text from the manuscript.

Reply: We updated the funding information of the online submission system with the relevant information. We further removed the funding statement from the text of the main manuscript.

3. Your ethics statement should only appear in the Methods section of your manuscript. If your ethics statement is written in any section besides the Methods, please move it to the Methods section and delete it from any other section. Please ensure that your ethics statement is included in your manuscript, as the ethics statement entered into the online submission form will not be published alongside your manuscript.

Reply: We now moved the ethics statement from the declaration section to the end of the Materials and Methods section of our manuscript.

Reviewer #1: TPLL is a rare disease with a very poor prognosis. This article identifies 3 subgroups of TPLL according to their transcriptional profile from data base. The manuscript is clearly written and structured.

Reply: We thank the reviewer for its positive feedback and valuable comments to our study.

Major Comments:

1. The manuscript lacks mechanistic data. Have there been verifications of the main candidate genes identified by another technique?

Reply: We apologize, but the main focus of our manuscript is on the computational analysis of T-PLL gene expression profiles with the goal to identify and characterize T-PLL subgroups based on publicly available data from the initial study by Schrader et al. (2018). We validated the subgroups with great success by hierarchical clustering in combination with bootstrapping and by independent validations on the basis of two other gene expression data sets from different gene expression platforms. Except for the newly added analysis of the correlation between the TCL1A protein and the *TCL1A* gene expression of T-PLL patients (see our answer to your point 6) based on the publicly available data by Schrader et al. (2018), we cannot include additional experimental validations of other genes.
Still, to further address this important point, we have now clearly written at the end of the Discussion section that additional experimental validations are necessary. It might for example be possible to reveal subgroup-specific pathomechanisms by gene perturbation experiments that target some of the predicted potential major regulators. Also analyses of drug screens in relation to the revealed subgroups could contribute to underline the potential clinical relevance of our findings.

2. There are duplicate patients between the training and validation series. They should be removed from the analysis.

Reply: To address this point, we have revised the validation Figure 7 and the corresponding section "Occurrence of the revealed T-PLL gene expression subgroups in other smaller patient cohorts" of the Results section. Now, Figure 7 additionally contains the subgroup distribution of the T-PLL patients from the RNA-Seq data set by the Herling lab that did not overlap with T-PLL patients from our microarray-based study. We further confirmed by Fisher's exact test that this subgroup distribution did not differ significantly from the subgroup distribution of our microarray-based study ($p = 0.23$). Thus, similar to the results for the completely independent T-PLL patients from Erkeland et al. (2022) also the cohort of non-overlapping T-PLL patients from the Herling lab represents the revealed subgroups in similar proportions as observed for our microarray-based cohort. This further supports the robustness of our findings and suggests that independent T-PLL patients also represent the three revealed subgroups.

3. Are the patients at diagnosis or in relapse? The table in the supplemental data seems to indicate more patients in follow-up. Specify the number and lines of treatment received. Could this recruitment heterogeneity have an impact on the molecular classification identified?

Reply: To address this point, we updated the meta-information of each included patient sample in S1 Table with the number and lines of treatment received and with the days between the sample acquisition and the end of the last treatment. We also added these pretreament information to the meta-information that we mapped to the T-PLL patients in the heatmap in Figure 1. Based on this new additional information, we were now able to report that for 12 of 68 patients (68 included in final analysis after exclusion of two outlier samples) the corresponding samples were taken in relapse after a previous therapy (median time of 120 days between sample acquisition and end of last treatment), whereas the samples of all other patients were from untreated patients at diagnosis. We now also checked how these 12 patients were distributed among our three revealed T-PLL gene expression subgroups. Each subgroup contained at least one of these pretreated patients (SG1: 4 of 18, SG2: 1 of 11, SG3: 7 of 39). There was no significant difference in the distribution of pretreated patients across the T-PLL subgroups (Fisher's exact test: $p = 0.75$). This means that the inclusion of pretreated patients did not strongly impact on the identification and the molecular composition of the revealed T-PLL subgroups. To further address this important point, we have reported these new additional findings in the subsection “Genome-wide gene expression analysis reveals three distinct T-PLL subgroups” of the Results section. We also briefly mention in the subsection “Gene expression data of T-PLL and normal control samples” of the Materials and Methods section that 58 T-PLL samples were taken from untreated patients at diagnosis and that 12 T-PLL samples were taken from patients in relapse after a previous therapy.

4. Hyperleukocytosis is not necessarily high. Was there a sorting of tumor cells or a study of the cell composition to avoid contamination by normal cells? Specify the exact nature of the normal T cell population of reference.

Reply: To address this point, we have revised the subsection “Gene expression data of T-PLL and normal control samples” of the Materials and Methods section to provide more details about the considered publicly available gene expression profiles. The gene expression profiles by Schrader et al. (2018) were measured on the basis of purified T-cells that were obtained from peripheral blood of T-PLL patients or healthy donors. These T-cells were derived by gradient centrifugation followed by magnetic-bead based cell enrichment reaching a purity of more than 95%. The normal T-cell population used as reference control were CD3+ pan T-cells. We report all these additional details in the Materials and Methods section and provide the reference to the initial manuscript by Schrader et al. (2018) for more details. We now also briefly report at the beginning of the first subsection “Genome-wide gene expression analysis reveals three distinct T-PLL subgroups” of the Results section that T-PLL tumor T-cells are compared to normal CD3+ pan T-cells.

5. What clinical interest could these identified signatures have?

Reply: To address this point, we have extended the Discussion section of the manuscript. We now briefly report that the three revealed T-PLL gene expression subgroups can have the potential to contribute to an improved molecular stratification of T-PLL patients. The revealed T-PLL subgroups could also be associated with different responses to treatments. We of course also mention that such potential associations have to be identified and validated by additional experimental studies. Still, such knowledge could provide an important basis for patient-specific treatment decisions.

6. How is the expression of TCL1a defined? Is there a correlation with GEP?

Reply: The protein expression of TCL1A was measured in Schrader et al. (2018) by flow cytometry in combination with antibodies against human TCL1A (clone 1-21) from BioLegend. To address this point, we used the available immunophenotypic TCL1A protein expression assignment of each T-PLL patient to one of the three classes (TCL1A protein expression status: positive, weak, negative) from Schrader et al. (2018) to associate the protein expression status of TCL1A with its corresponding gene expression level. The corresponding boxplots show a significant association between the protein expression status and the corresponding *TCL1A* gene expression level (Kruskal-Wallis test: $p = 0.0001238$). The median *TCL1A* expression levels of the T-PLL patients with positive TCL1A protein expression status were significantly greater than those of patients in the TCL1A negative status group (Wilcoxon rank sum test: $p = 0.0004$) or than those of patients in the TCL1A weak status group (Wilcoxon rank sum test: $p = 0.01$). To address this point, we have added these new findings to the subsection “Genome-wide gene expression analysis reveals three distinct T-PLL subgroups” of the Results section and refer to the newly created S3 Figure, which contains the corresponding boxplots along with information about the underlying data and how they were measured.

Reviewer #2: Thank you for the opportunity to review the manuscript entitled: “Computational gene expression analysis reveals distinct molecular subgroups of T-cell prolymphocytic leukemia” by Mikhaylenko et al. The study described the computational analysis of 68 gene expression profiles of one of the largest currently existing T-PLL patient cohorts. They identified three T-PLL subgroups: SG1, SG2 and SG3 that differed for genes that were either up-regulated or down-regulated. They also compared these profiles with putative normal counterparts. Differences between the three T-PLL subgroups were also observed at the level of signaling and metabolic pathways. Overall the study is well organized, clear in its structure and with some interesting conclusions. The analysis is rigorous and well-conducted. Figures are clear. The data were taken from Schrader A et al, Nat Commun. 2018 and thoroughly re-analyzed. While Schrader A et al also identified 4 groups (1 mostly normal samples, and 3 T-PLL groups), their analysis was not as deep as in the present manuscript. Thus, some novelty and new concepts are described in the present manuscript that could be useful for researchers working with T-PLL. Technically, there are not major

revision needed as the analysis are overall well-conducted.

Reply: We thank the reviewer for its very positive feedback to our manuscript.
